# Evaluation of Mithramycin in Combination with Chemotherapeutic Agents Against Ewing Sarcoma Cell Lines

**DOI:** 10.3390/cancers17182977

**Published:** 2025-09-11

**Authors:** Christoffer Briggs Lambring, Lina Albeer, Aneth Ochoa Negrete, Kayla Fure, Riyaz Basha

**Affiliations:** 1 Department of Microbiology, Immunology and Genetics, College of Biomedical and Translational Sciences, University of North Texas Health, Fort Worth, TX 76107, USA; 2 Texas College of Osteopathic Medicine, University of North Texas Health, Fort Worth, TX 76107, USA

**Keywords:** Ewing sarcoma, targeted therapy, combination index, vincristine, mithramycin, etoposide, apoptosis

## Abstract

Ewing Sarcoma (ES) is a rare, malignant bone neoplasm that is primarily diagnosed in childhood and adolescence. The aggressive nature of this neoplasm requires the use of surgery, radiation and a rigorous chemotherapy regimen. Metastatic ES carries a poor prognosis, which necessitates the development of new therapeutic agents. Mithramycin was tested in targeted therapy due to its specific inhibitory effects on the EWS-FLI1 fusion protein which is present in >85% of ES tumors. We tested the combination of Mithramycin with chemotherapeutic agents vincristine (VCR) and Etoposide (Eto) for inducing higher cytotoxicity against ES cells, CHLA10 and TC205. Cardiomyocyte cell line, H9C2 was used to test the effect on non-malignant cells. Cell viability was measured using the CellTiter-Glo kit, and the combination index was evaluated to determine the type of combination response (antagonistic, additive, or synergistic). Apoptotic cells were measured post-treatment with vehicle (DMSO, control), monotherapy (mithramycin or etoposide), or combination therapy (mithramycin + etoposide) using BD LSRII flow cytometer and analyzed utilizing FlowJo software V8.0. The apoptotic protein marker c-PARP in both treatment and control groups was analyzed using Western blot analysis. The results showed higher cytotoxicity for combination treatment when compared to individual agents, and the combination index confirmed the response as synergistic. H9C2 cells did not demonstrate significant decreases in cell viability when treated with combination therapy, highlighting the specificity of the treatment toward its target tissue. Flow cytometry confirmed the underlying mechanism as upregulation of apoptosis which is further supported by an increase in effector caspases 3/7 and elevated expression of c-PARP. These in vitro assays using ES cells provide preliminary evidence for the benefit of chemotherapy and mithramycin combination.

## Simple Summary

Ewing sarcoma primarily affects adolescents and young adults and is considered an aggressive malignancy with a poor prognosis. Mithramycin has been identified as a specific inhibitor of the EWS-FLI1 fusion protein, which is expressed in over 85% of patients with this cancer. However, its clinical application has been limited by toxicity. This study evaluated the anti-proliferative effects of Mithramycin in combination with the chemotherapeutic agents Vincristine and Etoposide. The results demonstrated a significant increase in cytotoxicity when Mithramycin was used together with chemotherapeutic agents compared to either agent alone. Further experiments were conducted to evaluate the response of Mithramycin and Etoposide combination on apoptosis. Flow cytometric analysis showed an upregulation of annexin-V-positive cells, and Western blot detection of cleaved PARP confirmed apoptosis activation. Notably, non-malignant H9C2 cardiomyocytes showed no significant reduction in proliferation with Mithramycin, Etoposide, or their combination. These findings provide preliminary evidence that Mithramycin can enhance the chemotherapeutic response in vitro. Further studies are underway to elucidate its precise mechanism of action.

## 1. Introduction

Ewing Sarcoma (ES) is the second leading cause of bone tumors in adolescents and children. It mainly affects the demographic of children and young adults from the age of 10 to 20 years old, with diagnoses most frequent in boys between 10 and 15 years old. ES is a tumor that typically develops in bone and soft tissues, particularly in long bones, the spine, ribs, and pelvis. Metastases are most commonly found in the lungs, bone, and bone marrow in up to 25% of patients at the time of diagnosis [1]. Pathologically, ES is classically characterized as a small round cell tumor. Some ES tumors have neural differentiation and can be referred to as primitive neuroectodermal tumors (PNETs). The tumors have characteristic Homer Wright Rosettes, which are cell clusters with neurofibrillary processes [2].

EWS-FLI1 fusion protein regulates oncogenes, including the ones associated with tumorigenesis [3]. This protein is expressed in >85% of ES tumors and the efficacy of Mithramycin (Mit) was tested as a targeted therapeutic agent to inhibit transcription of the EWS-FLI1 fusion protein in ES cells [4]. While mithramycin’s efficacy is evident in the compound’s inhibitory activity on the EWS–FLI1 protein, clinical application of this drug had proven to be challenging. In a previous clinical trial, both children and adults with ES received Mit alone, with average plasma concentrations of around 18 ng/mL [4]. At this concentration, many patients experienced severe elevations in alanine aminotransferase and aspartate aminotransferase, leading to discontinuation of treatment. Unfortunately, this plasma concentration was far below the required level to inhibit EWS-FLI1 transcriptional activity. The strong efficacy of Mit against ES prompts the question of how to utilize the gene-suppression activity of Mit and minimize toxicity due to drug exposure. Combination therapy of Mit and other chemotherapeutic agents provides not only a potential increase in treatment efficacy, but also the potential to lower the total dose of each individual agent and minimize toxicity.

The standard of care for ES includes combination induction chemotherapy followed by surgery and/or adjuvant chemotherapy [5,6]. In localized cases of ES, the combination of standard of care can lead to a 73% survival rate of patients, but the prognosis is poor in patients when ES has metastasized [2]. The current treatment for localized ES includes a combination of vincristine (VCR), Doxorubicin, and cyclophosphamide, with an alternating dose of ifosfamide and etoposide (Eto) every two weeks [2]. For patients with metastasized ES, the treatment includes VCR, doxorubicin, and cyclophosphamide, alternating with ifosfamide, and Eto every two weeks [2]. However, these combinatorial treatments were not reported to demonstrate improved survival of the patients or post-treatment quality-of-life. A previous clinical trial examined the efficacy of adding ifosfamide and Eto to the standard treatment regimen of VCR, doxorubicin, cyclophosphamide, and dactinomycin improved treatment outcomes. Patients with metastatic ES or PNET were treated with 9 weeks of chemotherapy before local control and then an additional 42 weeks of chemotherapy. After this regimen, some patients were treated with alternating ifosfamide and Eto. Following the clinical trial, the event-free survival was less than 30%, concluding that the addition of these agents did not increase survival outcomes; however, these agents remain a part of the standard treatment regimen [7]. Thus, finding new possible treatments that can be used as monotherapy or in combinational therapy is of utmost importance.

In recent years, there has been a growing interest in Mit’s inhibitory effects on the aberrant fusion of the transcription factor EWS-FLI1 that results from the chromosomal translocation from t (11;22) (q24;q12) [2]. This translocation results in the fusion of the constitutively expressed EWSR1 protein and the FLI1 protein. The EWSR1 protein plays a role in homologous recombination, which becomes impaired in ES due to the chromosomal translocation [8]. We hypothesize that using Mit in combination with chemotherapeutic agents will induce cytotoxicity of cancer cells through alternative mechanisms to inhibit the ESW-FLI1 fusion protein. To determine Mit’s effectiveness as a cancer therapeutic, cell viability testing was initially performed to identify proper dosages of monotherapy and combination use for Mit. Optimized doses were then tested in combination treatment to evaluate the effect on apoptosis.

## 2. Materials and Methods

### 2.1. Cell Culture

ES cell lines were obtained from Children’s Oncology Group (Lubbock, TX, USA) and H9C2 cells were purchased from ATCC (Manassas, VA, USA). CHLA-10 (EWS-FLI1-expressing) and TC205 (EWS-FLI1-non-expressing) cells, as well as cardiomyocytes (H9C2) were grown in Iscove’s Modified Dulbecco’s Media (10% FBS, 1% penicillin, 1% ITS, 4 mM glutamine, HEPES). Cell lines were grown at 37 °C and 5% CO_2_. 

### 2.2. Chemicals, Reagents, and Antibodies

Mithramycin, Dimethyl sulfoxide (DMSO), Eto, VCR and β-actin antibody were obtained from Sigma-Aldrich (St. Louis, MO, USA). Cleaved poly (ADP-ribose) polymerase (c-PARP) antibody was purchased from Cell Signaling Technology (Danvers, MA, USA) and PE-Annexin V/7-AAD apoptosis kit was obtained from BD Biosciences (Franklin Lakes, NJ, USA).

### 2.3. Cell Viability

ES cells were grown, and 4000 cells were then plated per well in 96-well plates. After 24 h they were treated with DMSO (control) or Mit, and/or Eto/VCR. Cells were incubated at 37 °C and 5% CO_2_, and after 48 h, cell viability was analyzed using a CellTiter-Glo kit (Promega, Madison, WI, USA). Following the protocol outlined in the CellTiter-Glo kit, 100 µL of CellTiter-Glo reagent was added to each well of the 96-well plates and left in the dark to incubate for 30 min. Luminescence (measures the cellular ATP, which is proportional to living cells) was recorded using a Synergy HT microplate reader. Each treatment and control was measured in triplicate, and data were graphed as percent viability against concentration on a log scale. IC50 values were calculated using GraphPad Prism software V9.0. Combination index was calculated using Calcusyn software V2.0 after treatments were set in a constant-ratio design and read using CellTiter-Glo (Promega, Madison, WI, USA) [9,10]. Data was analyzed using one-way ANOVA.

### 2.4. Flow Cytometry: PE Annexin-V Staining

ES cells were harvested after treatment with vehicle (DMSO), or drug alone (Mit or Eto), or combination (Mit + Eto) for 48 h. The apoptotic cell population was determined following standard procedure described by the manufacturer using PE-Annexin V/7-AAD apoptosis kit. BD LSRII flow cytometer was used to measure the staining and the data was analyzed (FlowJo software V8.0). Data was analyzed using one-way ANOVA.

### 2.5. Western Blot: C-PARP Expression

Protein was harvested after 48 h of treatments and a BCA assay was performed to find protein concentration. Western blot data were obtained using a capillary Western analysis Jess system from Bio-Techne (Minneapolis, MN, USA). Samples were run on 25 capillary cartridges, 12-230 kDa separation from Bio-Techne. A total of 1 mg of protein lysate was loaded onto the plate, followed by primary antibodies for c-PARP (1:50) or β-actin (1:100). Loading of the plate was performed according to manufacturer instructions and subsequent separation and immunodetection occurred in the automated capillary system. Blots were generated, and the area under the curves from the chromatogram data was analyzed using Compass for Simple Western software (version 6.1). Expression of all proteins was normalized to actin. Data was analyzed using one-way ANOVA.

## 3. Results

### 3.1. Dose Response and Determination of IC_50_ Values

**Figure 1:**
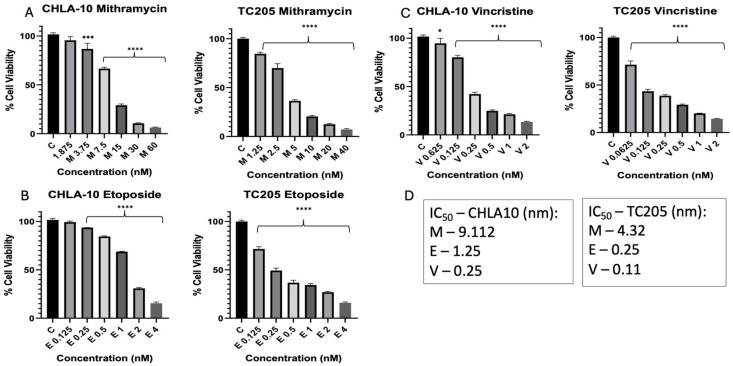
Monotherapy and IC_50_ values of CHLA-10 and TC205 ES cell lines. Cell viability results are shown for varying concentrations of monotherapy treatments of Mithramycin (Mit) and Etoposide (Eto) (**A**–**C**). M: Mithramycin; E: Etoposide; V: Vincristine. IC_50_ values per drug per cell line are shown (**D**). Viability data represented are all 48 h treatments. Data were analyzed using one-way ANOVA; **** *p* < 0.0001, *** *p* < 0.0002, * *p* < 0.02.

**Figure 2:**
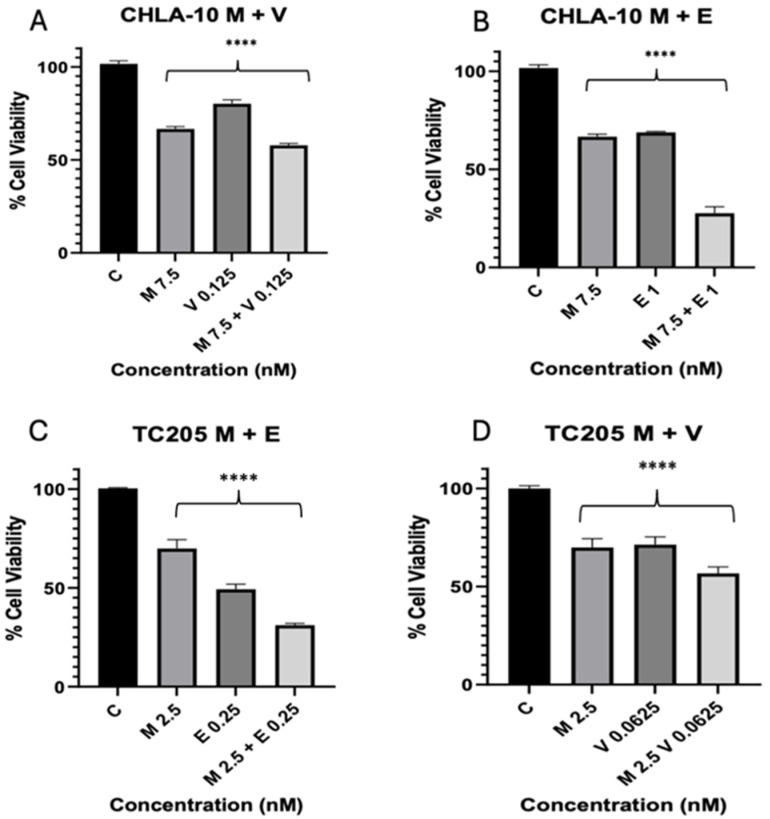
Combination treatments of CHLA-10 and TC205. ES cell lines treated with Mithramycin and Etoposide or Mithramycin and Vincristine (**A**–**D**). M: Mithramycin; E: Etoposide; V: Vincristine. Viability data represented are all 48 h treatments. Data were analyzed using one-way ANOVA; **** *p* < 0.0001.

**Figure 3:**
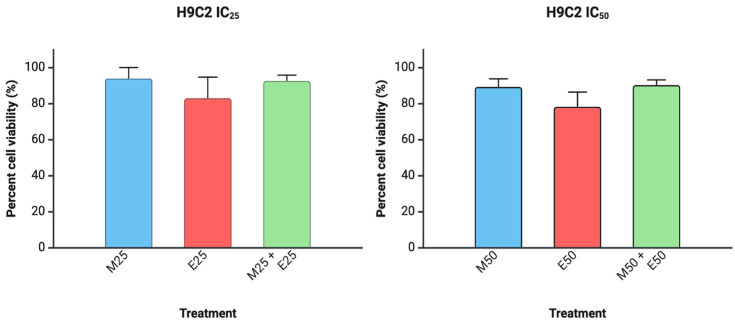
Treatment effects on cardiomyocyte cell line. H9C2 cells after treatments with IC_25_ and IC_50_ doses. Mithramycin, Etoposide, and Vincristine are indicated with M, E, and V, respectively. Viability data represent 48 h treatments. Data were analyzed using one-way ANOVA.

The antiproliferative effect of each drug was tested on two cell lines, TC205 and CHLA10. To establish working IC_50_ values, Mit, Eto, and VCR were analyzed over 48 h treatment periods, resulting in dose-dependent inhibition of cell viability. IC_50_ doses varied per drug and cell line (Figure 1). Combination treatments were also used at approximate IC_50_ values, resulting in improved reduction in cell viability for each combination tested (Figure 2). The IC_50_ values for CHLA10 IC_50_ values were 9.11, 1.25, and 0.25 nM for Mit, Eto, and VCR, respectively. For TC205, the IC_50_ values were 4.32, 0.25, and 0.11 nM for Mit, Eto, and VCR, respectively. Testing against non-malignant H9C2 cells (Figure 3) showed relatively little change in cell viability with Mit and Mit + Eto treatment. The only relatively greater change was observed with monotherapy IC_50_ Eto treatment (*p* = 0.055). Interestingly, an antagonistic response was observed with the Eto and Mit combination, which reduced the growth inhibition caused by Eto in H9C2 cells.

### 3.2. Combination Index

**Figure 4:**
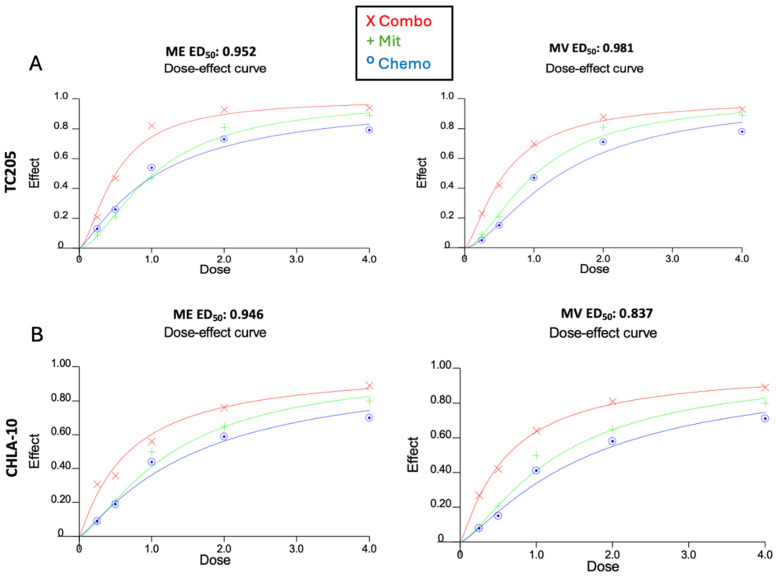
Combination index for CHLA-10 and TC205 ES cell lines. Combination treatments for TC205 (**A**) and CHLA-10 (**B**) with Mithramycin + Etoposide (ME) or Mithramycin + Vincristine (MV). ED_50_ values refer to effective dose 50; ED_50_ <1, =1, and >1 represent synergistic, additive, or antagonistic response, respectively.

To study further treatment interactions, drug effect analysis using Calcusyn provided ED50 values and representative dose-effect curves to determine whether the combination produced a synergistic, antagonistic, or additive response. Details of the combination index calculation were well established and described earlier [11]. Treatment concentrations were set in a constant-ratio design and analyzed using CellTiter-Glo (Figure 4). In both CHLA10 and TC205 Mit combinations with Eto and VCR exhibited ED50 values <1, indicating synergistic drug responses in each case [12].

### 3.3. Mithramycin and Eto Effects on Apoptotic Cell Population

**Figure 5:**
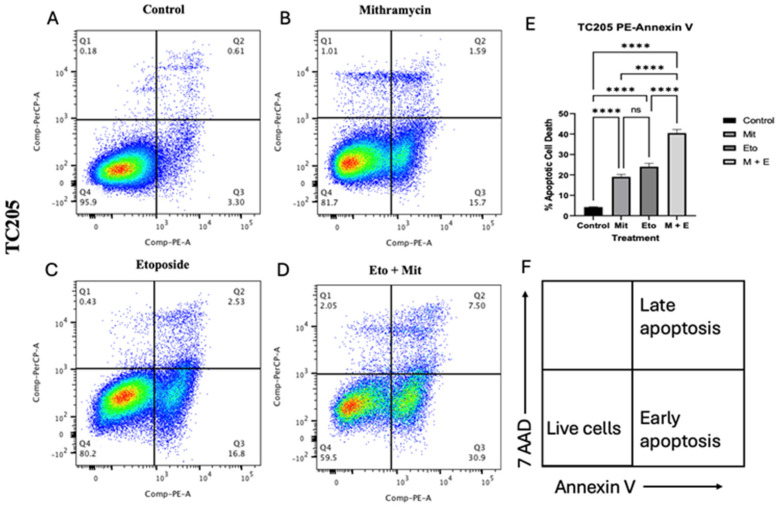
Effect of Mithramycin and Etoposide on apoptosis. Annexin V-PE/7-AAD flow cytometry plots and quantified results following 48 h treatment with IC_25_ doses for TC205 cells (**A**–**D**). M: Mithramycin; E: Etoposide; V: Vincristine. Quantification of percept apoptotic cell death (**E**). Representative diagram of apoptosis using Annexin V-PE/7-AAD staining (**F**). Flow cytometry plots show the density gradient in the cell populations, with red representing highest population density, and blue representing the lowest population density. Data were analyzed using one-way ANOVA; **** *p* < 0.0001.

Flow cytometric analysis of TC205 cells after 48 h treatments of Mit and Eto were conducted to identify separate apoptotic cell populations. Percent change in viable cells and early and late apoptotic cell populations illustrate monotherapy and combination treatment. A positive shift in apoptosis was observed in all treatments; however, the most effective results were seen in the combination groups (Figure 5). 

### 3.4. Cleaved PARP Expression

**Figure 6:**
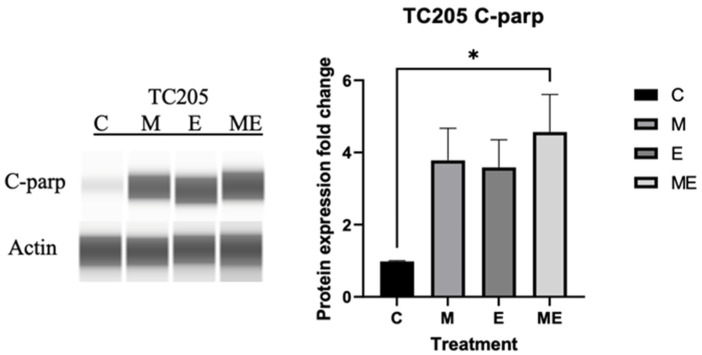
Effect of Mithramycin and Etoposide on c-PARP expression. c-PARP expression was measured via Simple Western Jess system, and quantification for TC205 protein following 48 h treatment with IC_25_ doses was obtained in GraphPad Prism from at least 3 independent runs. M: Mithramycin; E: Etoposide; ME: Mithramycin + Etoposide. Data were analyzed using one-way ANOVA, * *p* < 0.03.

Further apoptosis-related study through Western blot analysis of c-PARP in TC205 cells followed a similar trend, with the highest C-PARP expression occurring in the combination group (Figure 6).

## 4. Discussion

Chemotherapeutic drugs induce high levels of toxicity in ES patients [13,14,15,16]. Although novel therapies, including immunotherapy, are in clinical testing, standard treatment options involving chemotherapy and radiation remain the most commonly used in ES therapy [17,18,19,20,21]. The majority of cancer therapies are administered as combinations of drugs within broader treatment regimens. The combination of chemotherapeutic agents with less toxic agents to improve therapeutic efficacy has been employed in multiple studies [11,22,23,24]. In ES, it is particularly important to utilize a combinatorial treatment approach [25], especially when metastasis is detected in lungs or bone marrow [26,27,28]. Thus, testing the combination of Mithramycin (Mit) with Etoposide (Eto) or Vincristine (VCR) provides not only a more effective treatment option but also better reflects actual therapeutic implementation. 

The viability data of TC205 and CHLA-10 cells revealed that low-dose Mit, Eto, and VCR concentrations should be utilized for further studies. The calculated IC_50_ values from the cell viability assay were for the CHLA-10 cell line were 9.11, 1.25, and 0.25 nM for Mit, Eto, and VCR, respectively. For the TC205 cell line, the IC_50_ values were 4.32, 0.25, and 0.11 nM for Mit, Eto, and VCR, respectively. The IC_50_ values of Mit for the two ES cell lines tested in this study fall within the average range previously reported [29]. It has been reported that cell lines expressing the EWS-FLI1 aberrant protein are particularly sensitive to Mit [29]. The reported plasma levels in the ES clinical study were approximately 18 ng/ml [4]. In our study, we used 2.71 and 8.74 ng/mL, respectively, for TC205 and CHLA10 cells (calculated based on molecular weight and concentration). Since binding to serum proteins and absorption due to combination treatment can also influence the therapeutic effect, these doses are reasonable to test.

The results reported here show that TC205 was more sensitive than CHLA-10 to Mit at different concentrations, supporting the high sensitivity to Mit in inhibiting the EWS-FLI1 fusion gene (Figure 1A) [30]. Similarly, the results for the IC50 of Eto in this study show that lower dosages of Eto are needed for the TC205 cell line when compared to the CHLA-10 cell line (Figure 1B). Prospects for improving current therapies have been evaluated by comparing the inhibitory response of chemotherapeutic drugs, with average reported IC_50_ for Eto usually ranging from 0.30 µM to 1.11 µM depending on the specific ES cell line being tested [31,32,33]. Following the same pattern, the IC_50_ value for VCR was lower in the TC205 cell line compared with the CHLA-10 cell line (Figure 1C). The IC_50_ values for the ES cell lines tested in this study fall within the average IC_50_ values reported in previous studies. These low numbers show that VCR is effective at inhibiting the cell proliferation of ES [34].

To further improve the dosage utilized for treatment, a combinatorial therapy approach was investigated by combination dosing of Mit with Eto, as well as Mit with VCR. The combination dosing showed a decrease in viability in each instance of Mit + Eto and Mit + VCR when compared with monotherapy results (Figure 2A–D). Testing the safety of treatment doses against cardiomyocytes (H9C2) revealed minimal effect of Mit on non-malignant cells (Figure 3). In the combination, there was also slight recovery of H9C2 cells compared with Eto monotherapy, which warrants further study (Figure 3). To further explain the effective viability reduction seen in combination, we aimed to describe the drug interaction between Mit, Eto, and VCR. In both CHLA-10 and TC205 cells, combination index analysis showed a synergistic relationship (ED_5_ < 1) (Figure 4A,B). This synergistic relationship may be explained by the complementary behavior of their individual mechanisms.

Mithramycin inhibits the EWS-FLI1 fusion transcription factor by suppressing its transcriptional activity and altering downstream gene expression critical for tumor growth and DNA repair processes [29]. Some of the genes downregulated by Mit’s inhibition of DNA repair mechanisms can effectively block double-stranded DNA repair in the target cells, increasing the DNA damage in the cell resulting in increased apoptosis [35]. The cytotoxic effects of Eto are due to its inhibition of DNA topoisomerase II through stabilization of the DNA-topoisomerase II complex causing increased DNA breaks and damage, ultimately leading to increased cell death [31]. The combination of Mit with Eto suppresses DNA repair pathways via Mit, and the increase in DNA breaks caused by Eto further enhances the activation of apoptotic pathway initiators [31,34]. 

VCR can disrupt the formation of microtubule polymers during mitotic spindle assembly, resulting in the arrest of metaphase and increasing the cell death of ES dividing cells [34]. The use of vinca alkaloids, such as VCR, has been part of cancer treatment since the early stages of development, due to their M-phase-specific anti-tubulin capabilities [36]. However, the toxic effects of this family of alkaloids can be broad and affect multiple organ systems; therefore, treatment with VCR is typically performed in combinatorial treatments to achieve a lower effective dose [36]. The combination of Mit with VCR increases apoptosis through the downregulation of DNA repair mechanisms through Mit and the cell-cycle arrest caused by VCR-mediated disruption of microtubule formation.

To explain the decrease in viability of ES cell lines, we observed a marked increase in early and late apoptotic cells by flow cytometry of TC205 cells after treatment, with the combination dose offering the most significant impact on the apoptotic cell population (Figure 5). It has been reported that ES cells are particularly sensitive to inhibition of PARP, as the EWS-FLI1 oncoprotein interacts with PARP leading to increased transcriptional activation by EWS-FLI1 [37]. Treatments that target the DNA repair mechanisms can promote apoptosis through cleaving of PARP, generating the apoptosis effector protein c-PARP [38]. Western blot analysis of c-PARP, used as another identifier of apoptotic cells, showed a significant increase in all groups compared with the control group. However matching the flow cytometry data, the combination group had the highest expression of c-PARP (Figure 6). The increased expression of c-PARP confirms the flow cytometry results, showing that Mit, Eto and Mit with Eto inhibit DNA repair mechanisms in the cell, resulting in increased apoptosis.

This study has some limitations and we are continuing investigation to fulfill the gaps. It is still in the preliminary stage and limited to in vitro assays. Further validation using in vivo models is needed. Even though, cardiotoxicity with Eto is rare, it remains a serious concern especially in combination treatments [39,40]. Therefore, in this study we tested cytotoxicity against cardiomyocytes. Since Mit can affect liver, testing hepatotoxicity, along with comprehensive pharmacokinetic evaluations, will also be helpful to determine the therapeutic potential and safety profile of the combination. Other issue is using the concentration of Mit for laboratory testing aligning with the plasma levels of clinical relevance. While we show that the apoptotic process is elevated in ES cells due to treatment, the underlying molecular mechanisms responsible for this effect, particularly in the context of combination therapy, remain unclear. It is possible that multiple pathways contribute to the observed cytotoxicity. As we continue to evaluate this strategy, we will conduct experiments in detail to address outstanding issues.

## 5. Conclusions

Notably, there has been little improvement in treatment strategies or survival rates of ES patients over several decades; however, a few novel approaches are still in testing [41,42,43]. Using Mit, an older drug, this study provides evidence that it can enhance cytotoxicity in ES cell lines, CHLA-10 and TC205. While Mit has previously been studied for its specific inhibitory effect against EWS-FLI1 fusion protein, our findings suggest efficacy in both EWS-FLI1—expressing (CHLA-10) and non-expressing (TC205) cells. The results showed an increase in apoptosis; however, the precise molecular mechanisms responsible anti-proliferative response in Ewing sarcoma cells, particularly in combination therapy, remain unclear. Further investigations are needed to unravel underlying pathways and optimize its translational application.

## Data Availability

The data presented in this study are available in the article. Further inquiries can be directed to the corresponding author.

## Abbreviations

The following abbreviations are used in this manuscript:

c-PARP	Cleaved poly (ADP-ribose) polymerase (PARP)
DMSO	Dimethyl sulfoxide
DNA	Deoxyribonucleic acid
ED	Effective dose
ED_50_	Median effective dose
ES	Ewing sarcoma
Eto	Etoposide
IC_50_	Inhibitory concentration 50
M	Molar
Mit	Mithramycin
PARP	Poly (ADP-ribose) polymerase
PNET	Primitive neuroectodermal tumor
VCR	Vincristine
